# Effect of Barley and Oat Consumption on Immune System, Inflammation and Gut Microbiota: A Systematic Review of Randomized Controlled Trials

**DOI:** 10.1007/s13668-024-00543-x

**Published:** 2024-05-24

**Authors:** María-Engracia Cortijo-Alfonso, María-Paz Romero, Alba Macià, Silvia Yuste, Marian Moralejo, Laura Rubió-Piqué, Carme Piñol-Felis

**Affiliations:** 1 University of Lleida-Agrotecnio CERCA Center, Av. Alcalde Rovira Roure 191, 25198 Lleida, Spain; 2 Department of Medicine and Surgery, University of Lleida, Lleida, Catalonia Spain; 3 Institut de Recerca Biomèdica de Lleida, Fundació Dr. Pifarré IRBLleida, Lleida, Catalonia Spain

**Keywords:** Barley, β-glucans, Gut microbiota, Immune function, Inflammation, Systematic review, Oats

## Abstract

**Purpose of Review:**

The aim of this systematic review was to investigate the effects of whole grain *Avena sativa* and *Hordeum vulgare* L., or their isolated fractions, on immune and inflammatory functions, as well as their influence on gut microbiota. A structured literature search was undertaken in line with PRISMA guidelines. Randomized controlled trials (RCTs) that investigated the effects of oats or barley consumption in adults and reported ≥ 1 of the following: C-reactive protein (CRP), tumor necrosis factor (TNF-α), interleukin-6 (IL-6), IL-2, IL-8, IL-18, lipopolysacharide binding protein (LBP) or gut microbiota-related outcomes, were included.

**Recent Findings:**

A total of 16 RCTs were included, among which 6 studies recruited metabolically at-risk population, including individuals with overweight and obesity, metabolic syndrome or hypercholesterolemia. Additionally, 3 trials involved young healthy population, 5 trials targeted older individuals (aged over 50 years), and 2 studies encompassed populations with other disease states. A total of 1091 individuals were included in the evaluation of short-term (up to 14 days) and long-term (beyond 14 days, up to 90 days) supplementation with oats or barley-based products. 9 studies measured inflammatory biomarkers and 5 of them reported significant reductions, specifically in long-term studies. Notably, no evidence of anti-inflammatory benefits was found in healthy individuals, whereas studies involving metabolically at-risk populations showed promising reductions in inflammation. 13 studies measured the impact on gut microbiota, and collectively suggest that oats and barley food products can influence the composition of gut microbiota, associated in some cases with metabolic improvements.

**Summary:**

Oats and barley consumption may confer anti-inflammatory effects in metabolically at-risk populations and influence gut microbiota outcomes. However, no anti-inflammatory benefits were observed in healthy individuals. Results from this systematic review suggests caution in interpreting findings due to limited trials and variations in interventions and health conditions.

## Introduction

There is a growing body of evidence linking regular consumption of whole grain cereals with overall health benefits, specifically, with reduced risks of chronic cardiovascular diseases [1]. Among these cereals, whole grain oats and barley stand out for their rich content of soluble dietary fiber, notably β-glucans, and phytonutrients such as (poly)phenolic compounds [2]. While systematic reviews of randomized controlled trials (RCTs) have extensively explored the cardiovascular and glucose regulation benefits of barley and oats consumption [3–6], limited research has focused on their impact on inflammation and immune function. Notably, a narrative review [7•] delved into the components of oats, highlighting the potential implications for immune-related disorders. β-glucans found in oats have been shown to enhance innate immune responses and possess anti-inflammatory properties [8]. Furthermore, antioxidants present in oats, including phenolic compounds and vitamin E, have demonstrated the ability to mitigate inflammation and oxidative stress, thereby supporting immune function [9]. Additionally, vitamins such as B6, folate, and A, abundant in oats, play pivotal roles in antibody synthesis, lymphocyte proliferation, and mucosal integrity, respectively [10]. Overall, these oat components modulate immune responses and may have implications for immune-related disorders. However, there is still a gap in the literature regarding a comprehensive overview of studies specifically investigating the effects of barley and oats on inflammatory markers and gut microbiota in adult populations through human clinical trials.

Barley (*Hordeum vulgare* L.) is one of the most cultivated cereals in the world after maize, wheat and rice and is a highly adaptable crop grown in a wide diversity of climatic conditions [11]. In addition, it is rich in bioactive compounds such as dietary fiber and phytochemical components, including phenolic acids, flavonoids, tocols, lignans, phytosterols, and folates [12, 13]. Thus, barley grains are considered to have a very high functional value among cereal crops [14]. Although the health benefits of barley grain are very interesting, it is rarely used for human consumption, except for the brewing industry [15].

Oats (*Avena sativa* L.) have been cultivated for more than 2000 years in various regions throughout the world. It is a multifunctional crop considered to be nutritionally superior to many other unfortified cereals. Oats are commonly consumed as whole grains, which provide important nutrients such as proteins, unsaturated fatty acids, vitamins, and minerals [16]. Oats are also a good source of soluble dietary fiber, especially β-glucan, and contain numerous bioactive phytochemicals such as phenolic acids, flavonoids, vitamin E, phytosterols, avenanthramides and steroidal saponins [17].

In terms of health, several studies have shown the positive effects of barley and oats on glycemic index, cholesterol, and heart disease [18]. This is mainly due to the presence of β-glucan (2–11% d.w.), a component of dietary fiber with a health claim approved by the US Food and Drug Administration and the EFSA [3, 4, 19•]. Other beneficial effects of barley and oats based products have recently been described, such as the ability to modulate the composition and activity of the human gut microbiota [5]. Several studies have shown that gut microbiota is related to the occurrence and development of various diseases [6]. Moreover, in recent years, β-glucans have received considerable attention as immune system enhancers against infectious diseases and some types of cancer [20]. Chronic inflammation triggered by immune disorders is the central part of the pathophysiology of various non-communicable diseases, and dietary fiber intake is inexorably linked to the gut microbiome leading to a reduction in inflammation [21].

Barley and oats are also associated with beneficial health effects due to the presence of fiber-bound (poly)phenolic compounds. Whole grain barley and oats contain high levels of phenolic acids and flavan-3-ols, which can be found free, but they are predominantly covalently bound to the fibers [22], becoming available for uptake by the colonic microbiota. Up to 90% of the (poly)phenolic compounds ingested from whole grains are metabolized by the gut microbiota [23], which is able to release (poly)phenolics from fibers and metabolize them into more bioavailable bioactive compounds. Preclinical studies and clinical trials have shown that (poly)phenols from whole grains may play a crucial role in reducing the risk of pathologies associated with subclinical inflammation through the gut microbiota [24].

The aim of the present systematic review was to summarize the results of the RCTs evaluating the functionality of whole-grain barley and oats cereals or their isolated fractions on the immune response, the inflammatory biomarkers, and the gut microbiota modulation in adults.

## Materials and Methods

This systematic review of the RCT literature has been carried out following the Preferred Reporting Items for Systematic Reviews and Meta-Analysis guidelines (PRISMA).

### Elegibility and Exclusion Criteria

The research question when searching for information was "Is there an effect of barley/oat consumption or their isolated bioactive compounds on the immune system, inflammation or the gut microbiota?”.

Publications needed to meet the following inclusion criteria in the case of humans: a) RCT, parallel or cross-over design in humans aged > 18 years; b) without gender bias; c) without the presence of pregnant women, lactating women, chronically medicated or treated with antibiotics in the month prior to the study; d) avoidance of smokers or people who usually drink alcohol; e) studies with interventions that included whole grain barley, oat, or isolated extracts from these cereals; f) report ≥ 1 of some of the following outcomes: interleukin-6 (IL-6), IL-2, IL-8, IL-18, C-reactive protein (CRP), tumor necrosis factor (TNF), lipopolysaccharide binding protein (LBP), or gut microbiota composition.

Therefore, as exclusion criteria we would have those studies that did not meet the inclusion criteria set forth above.

The text included in the manuscript provides a clear list of search terms used in the systematic search. However, it can be improved by providing more context on how these terms were utilized in combinations to enhance the search strategy. Here's a revised version:

For the systematic search, we employed a comprehensive set of search terms, including "immune system", "barley", "Hordeum vulgare", "β-glucan", "beta glucan", "microbiota", "immune", "inflammation", "inflammatory", and "oat". These terms were systematically combined in pairs to enhance the search strategy and to ensure a thorough exploration of relevant literature.

### Search Strategy

The following online databases were searched: Medline and Cochrane Central Register of Controlled Trials (CENTRAL). Available online: https://ovidsp.ovid.com/ (Accessed December 13, 2021) and CINAHL. Available online: https://www.ebsco.com/ (Accessed December 13, 2021), from database inception through August 31, 2021. In addition, the reference lists of the eligible studies and PubMed were scanned. Available online: https://pubmed.ncbi.nlm.nih.gov/ (Accessed October 14, 2022).

### Study Selection, Data Extraction and Quality Assessment

Two reviewers, who assessed the full texts of potentially eligible studies, independently evaluated titles and abstracts. Two reviewers independently extracted the relevant information using a predefined data extraction form. Any disagreement between the reviewers was settled by reaching a consensus or by consulting a third reviewer.

A data extraction form was created in a Microsoft^®^ Excel^®^ spreadsheet (Microsoft 365 MSO Version 2109.14430.20306 Redmond, WA, USA) to facilitate the retrieval and storage of relevant data.

### Assessment of Risk of Bias of Individual Studies

The included studies were reviewed for risk of bias using the Cochrane Risk of Bias tool (Rob2) for RCTs [25]. Studies were assessed to determine if each study had a low, some concerns, or a high risk of bias. Assessment criteria included risk of bias arising from recruitment of subjects, randomization process, deviations from the interventions, missing data, measurement of outcome, or selection of the reported result.

In addition, to mitigate publication bias and ensure a balanced representation of the available literature, we adopted an inclusive approach, considering studies regardless of statistical significance or outcome direction, to encompass a broad spectrum of evidence, including positive and null findings.

## Results

### Search Results and Study Selection

**Fig. 1:**
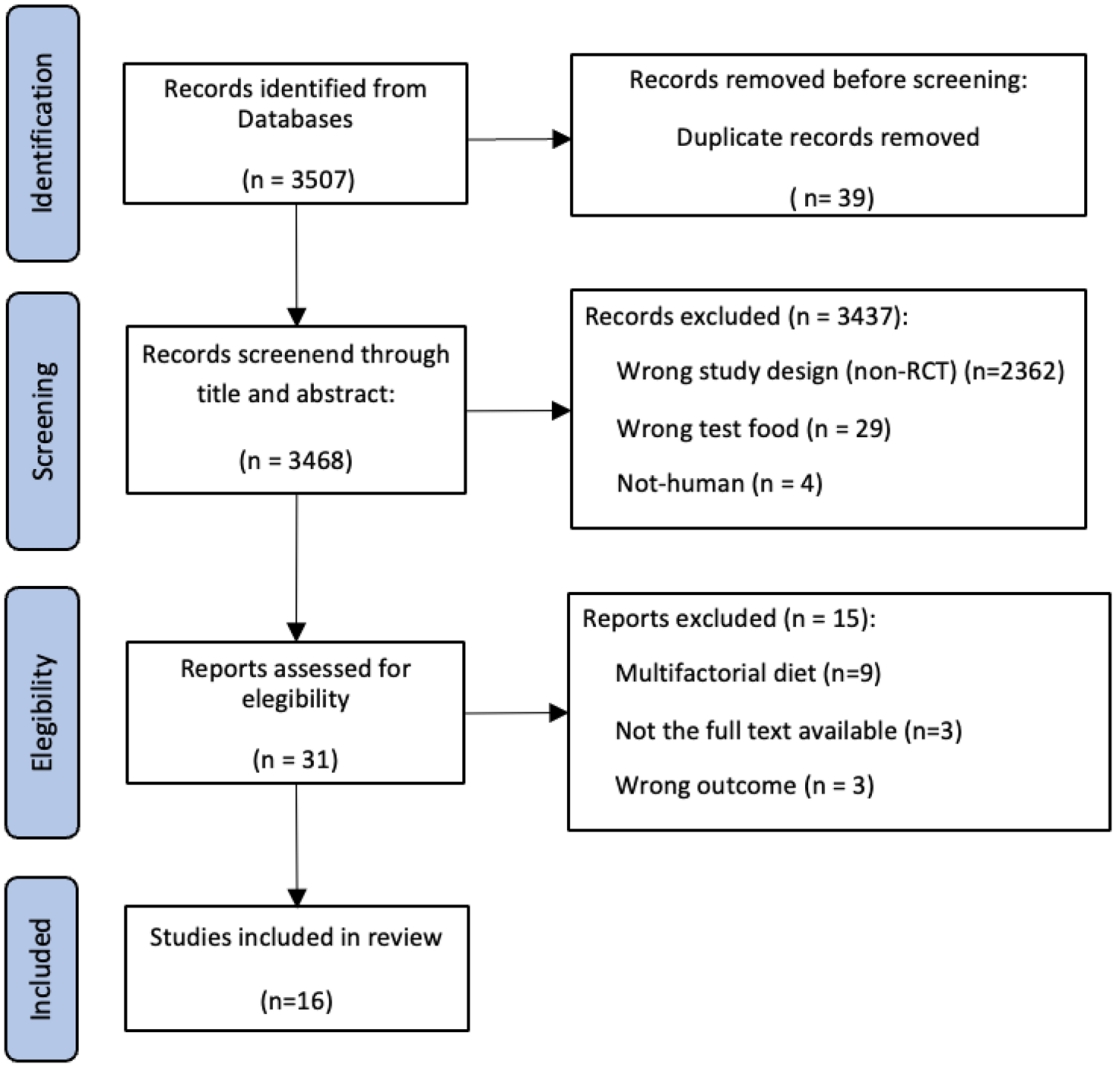
Preferred Reporting Items for Systematic Reviews and Meta-Analysis (PRISMA) flow diagram for study selection

The search strategy in databases revealed 3507 records in total. After removing 39 duplicates, 3468 records were identified for screening through titles and abstracts. Of these, 3437 references did not meet the inclusion criteria due to the wrong study design (non-RCT), did not contain the appropriate test foods/extracts (β-glucans from other sources such as mushrooms, yeast, or algae), or were non-human studies. Of the 31 records assessed for eligibility, 15 were excluded due to multifactorial diet, did not measure the target biomarkers, or unavailability of the full text. A remaining total of 16 RCTs were included in the systematic review (Fig. 1).

### Characteristics of Included Studies

Out of the 16 studies reviewed, 7 were parallel RCTs, and the remaining 9 were crossover RCTs, with a subset of them being either single or double-blinded. 4 studies comprised whole grain interventions, and 12 studies tested isolated β-glucan extracts from barley or oat. Records were additionally categorized into interventions involving barley (10 studies) or oats (6 studies), with one study incorporating both grains as the tested products [26].

Three studies were performed in healthy young adult population [27–29], 5 studies in older adults population (50–80 years old) [30–34], 6 studies in overweight, obese, or metabolically at risk population [26, 35–39], and 2 studies in populations with other disease states such as chronic gastritis and polypectomized patients [40, 41]. The studies had a total of 1091participants (mean of 68), with a mean age of 52,15 (range 23–70 years old) and the mean duration of the study was 4,83 weeks (range 1–12 weeks). 4 studies consisted of very short-term dietary interventions [26, 27, 31, 32]. Table 1 presents the characteristics of the included studies.

**Table 1:** Characteristics and main results of studies examining barley or oat derived products consumption on immune and inflammatory markers and gut microbiota

**Study**	**Country**	**design and duration**	**Subjects characteristics**	**N (M/F)**	**Age (years)**	**Intervention Diet (I)**	**Control Diet (C)**	**Outcomes**	**Background diet**	**Main results**
Nilsson et al. (2016) [27]	Sweden	Crossover 4 days	Healthy young adults	21(3/18)	23.9 ± 0.7	Barley kernel bread (75% barley kernel) 256 g bread/day	148 g/day white wheat bread	IL-6, IL-18, CRP, s-PAI-1, H_2_ levels in expired breath	Maintain habitual diet	↔ concentrations of s-IL-6, s-IL-18, s-PAI-1 or s-CRP at fasting or during the experimental day after I compared to C
										I ↑ H_2_ concentrations compared to C indicating increased gut microbiota activity
Martínez et al. (2013) [28]	USA	Crossover 4 weeks	Healthy young adults (normoweight and overweight)	28 (11/17)	25.9 ± 5.5	G1: 60 g/day of whole grain barley flakes (WGB)	-	LBP, hs-CRP, IL-6, IL-18 and fecal microbiota composition	Maintain habitual diet	After all treatments ↓ plasma IL-6, especially after BR+WGB
						G2: 30 g of brown rice (BR) + 30g of WGB				WGB, BR+WGB and BR ↑ the Firmicutes/Bacteroidetes ratio, and the abundance of the genus *Blautia*. Only WGB ↑ the genera *Roseburia*, *Bifidobacterium* and *Dialister*, and the species *Eubacterium rectale*, *Roseburia faecis* and *Roseburia intestinalis*
						G3: 60 g/day of BR				
Aoe et al. (2018) [29]	Japan	Parallel, double-blind 4 weeks	Healthy adults	60 (19/41)	46.5 ± 8.2	G1: 12 g/day of wheat bran cereal bars (WB)	40.7 g of cereal bars with neither wheat bran nor barley	Fecal microbiota composition, fecal SCFA	Maintain habitual diet	WB ↑ fecal SCFA and ↑ the abundance of butyrate-producing bacteria WB + BM ↑ in the abundance of the genus *Bacteroides*
						G2: 12 g/day of barley cereal bars (BM)				
						G3: 12 g of WB + 12g of BM				
Goto et al. (2022) [30]	Japan	Crossover, double-blind, 4 weeks	Older adults	19 (10/9)	53.8 ± 2.6	150 g of multi grain rice with pearled barley (22 g barley/day)	150 g/day of barley-free multi grain rice	Fecal microbiota composition	Substitute the staple food in their diet with the test or control food twice a day	I ↑ relative abundance of *Blautia, Agathobacter,* and *Fusicatenibacter*, compared to baseline
Nilsson et al. (2015) [31]	Sweden	Crossover 3 days	Older adults	20 (3/17)	64.1 ± 5.9	338 g/day of barley kernel bread (85% barley kernels)	338 g/day of white wheat bread	s-SCFA, s-IL-6, s-IL-18, breath H_2_ excretion	Standardize their meal, avoid alcohol, exercise, or foods rich in DF	Breath H_2_ excretion and total s-SCFA ↑ after I compared with C
										↔ s-IL-6, s-IL-18 after I compared to C
Sandberg et al. (2019) [32]	Sweden	Crossover 3 days	Older adults	99 (25/74)	64.1 ± 5.6	115 g/day of barley kernel bread (85% barley kernels)	103 g/day of white wheat bread	IL-6, CRP, breath H_2_ excretion	Maintain habitual diet	I ↑ breath H_2_ excretion compared to C
										I ↓ IL-6 compared to baseline only in the highest Prevotella/Bacteroides ratio subgroup
Velikonja et al. (2019) [35]	Slovenia	Parallel, double-blind 4 weeks	With risk or diagnosed with metabolic syndrome	43 (10/33)	50.93 ± 7.4	200 g/day wheat bread with barley β-glucans (6% β-glucans)	200 g/day of wheat bread	Faecal microbiota composition	Maintain habitual diet	I ↓ in 25% in total cell numbers of *Clostridium leptum* compared to baseline
										↔ among the other examined bacterial groups
Sawicki et al. (2016) [26]	USA	Crossover, double-blind 3 days	Overweight or mildly obese, metabolically at-risk and postmenopausal women	13 (8/5)	53.1 ± 7.0	G1: whole oat flour muffin. 48.7 g oat/day G2: whole barley flour muffin. 48.7 g barley/day	48.7 g/day of refined wheat flour plus cellulose	hsCRP, IL-6, IL-8, TNF-α	Avoid high amounts of alkylresorcinols, phenolic acids, phytosterols, tocols for 2 days prior	↔ of s-IL-6, s-IL-18, TNF- α, hsCRP after I (oat and barley) compared to C
			(BMI = 32.3 ± 3.1 kg/m^2^)							
Wang et al. (2016) [36]	USA	Crossover, single-blind 5 weeks	Mildly hypercholesterolemic	30 (12/18)	59 (55- 63)	G1: 3 g/day of low molecular weight barley β-glucan (LMB)	Wheat and rice based breakfast	Fecal microbiota composition	American Heart Association diet (AHA). Lunch and dinner were designed using a 7-day rotating menu	HMB ↑ Bacteroidetes and ↓ Firmicutes abundances compared to C HMB ↑ *Bacteroides*, tended to ↑ *Prevotella* and ↓ *Dorea* ↔ in the gut microbiota composition after LMB compared to C
						G2: 5 g/day of LMB/day				
						G3: 3 g/day of high molecular weight barley β-glucan (HMB)				
Turunen et al. (2010) [41]	Greece	Cross-over, double-blind 90 days	Polypectomized patients	20 (10/10)	57.6 (51–68)	125 g/day of wheat bread enriched with barley β-glucan (3 g of β-glucan)	125 g/day of commercial wheat bread	Fecal microbiota composition, fecal SCFA	Maintain habitual diet	I ↓ total coliform counts in feces compared to C
										↔ of other bacterial microbiota after I compared to C
										↔ of fecal SCFA after I compared to C
Ganda Mall et al. (2015) [33]	Sweden	Parallel 6 weeks	Older adults (with non-steroid anti-inflammatory treatment)	49 (27/22)	69.5 ± 7	12 g/day of oat β-glucan	12 g/day of maltodextrin	CRP, IFN-y, IL-10, IL-6, IL-8, TNF- α, CRP, fecal microbiota composition	Maintain habitual diet	↔ of inflammatory cytokines after I compared to C ↔ of microbiota composition after I compared to C
Laue et al. (2013) [34]	Germany	Parallel, double-blind, 5 weeks	Older adults (under influenza vaccination)	239 (121/118)	67.9 (50–79)	10 g/day of oat β-glucan	12 g/day of maltodextrin	IFN, TNF, IL1B, IL2, IL12, IL10, fecal microbiota composition and SCFA	No pro/prebiotics or fermented products, vitamins and minerals and a low dietary fiber diet	I ↑ IFN-γ levels between baseline and one week after vaccination compared to C ↔ of fecal SCFA after I compared to C
										↔ TNF, IL1B, IL2, IL12, IL10 over time and between I and C
										I ↑ *Parasutterella* relative abundance compared to C
El Hadji et al. (2022) [39]	USA	Parallel double-blind 4 weeks	Moderate-to-elevated LDL-cholesterol	191(73/118)	48 (21- 65)	3 g/day of oat β-glucan powder	Rice powder control	Systemic chronic inflammation (iAge)	Usual dietary, refrain from consuming oat, barley and psyllium products	I ↓ systemic chronic inflammation in a subset of subjects presenting elevated risk factors compared to C
Xu et al. (2021) [37]	China	Parallel single-blind 45 days	Mildly hypercholesterolemic	187 (129/28)	49.3 ± 11.0	80 g/day of oat (3 g β-glucan)	Rice control	Fecal microbiota composition	Maintain habitual diet	Oat ↑ *Akkermancia muciniphila Roseburia*, *Bifidobacterium* and *Faecalibacterium prausnitzii* compared to baseline
Pavadhgul et al. (2018) [38]	Thailand	Cross-over 4 weeks	Hypercholesterolemic	24	Between 30–60 years	70 g/day of oat flakes made with 100% whole grain oat (3.36 g β-glucans)	Instant white rice flakes	hs-CRP, TNF-α, IL-6, IL-8	Avoid oat or β-glucan and antioxidant supplements one week before	I ↓ hsCRP, IL-6, IL-8, and TNF-α compared to C and compared to baseline
Gudej et al. (2021) [40]	Poland	Parallel, double-blind 4 weeks	Diagnosed chronic gastritis	48 (34/14)	52.4 ± 3.5	G1: 3 g/day of high molar mass β-glucan solution G2: 3 g/day low molar mass β-glucan solution	100 mL solution of potato starch	TNF- α, CRP, fecal SCFA, number of LAB	Not fiber supplements or parapharmaceuticals	After G2 ↓ CRP compared to its baseline ↔ of TNF- α after G1 or G2 ↔ of LAB after G1 or G2 After G1 and G2 ↑ acetic acid, propionic acid and hydroxybutyric acid in feces

*N* Number of participants, *I* Intervention, *C* Control, *M* Male, *F* Female, *G* Group, *hs-CRP* high sensitivity C-Reactive Protein, *s-PAI-1* s-Plasminogen Activator Inhibitor, *TNF- α* Tumor Necrosis Factor, *LBP* Lipopolysaccharide-Binding Protein, *SCFA* short chain fatty acids, *IFN* Interferon gamma, *WGB *Whole grain barley, *BR* Brown Rice, *BMI *Body Mass Index, *DF* Dietary Fiber, *WB *Wheat Bran, *BM* Barley Cereal Bars, *EPS* Bacterial exopolysaccharide, *LAB *Lactic Acid Bacteria

### Risk of Bias

**Fig. 2:**
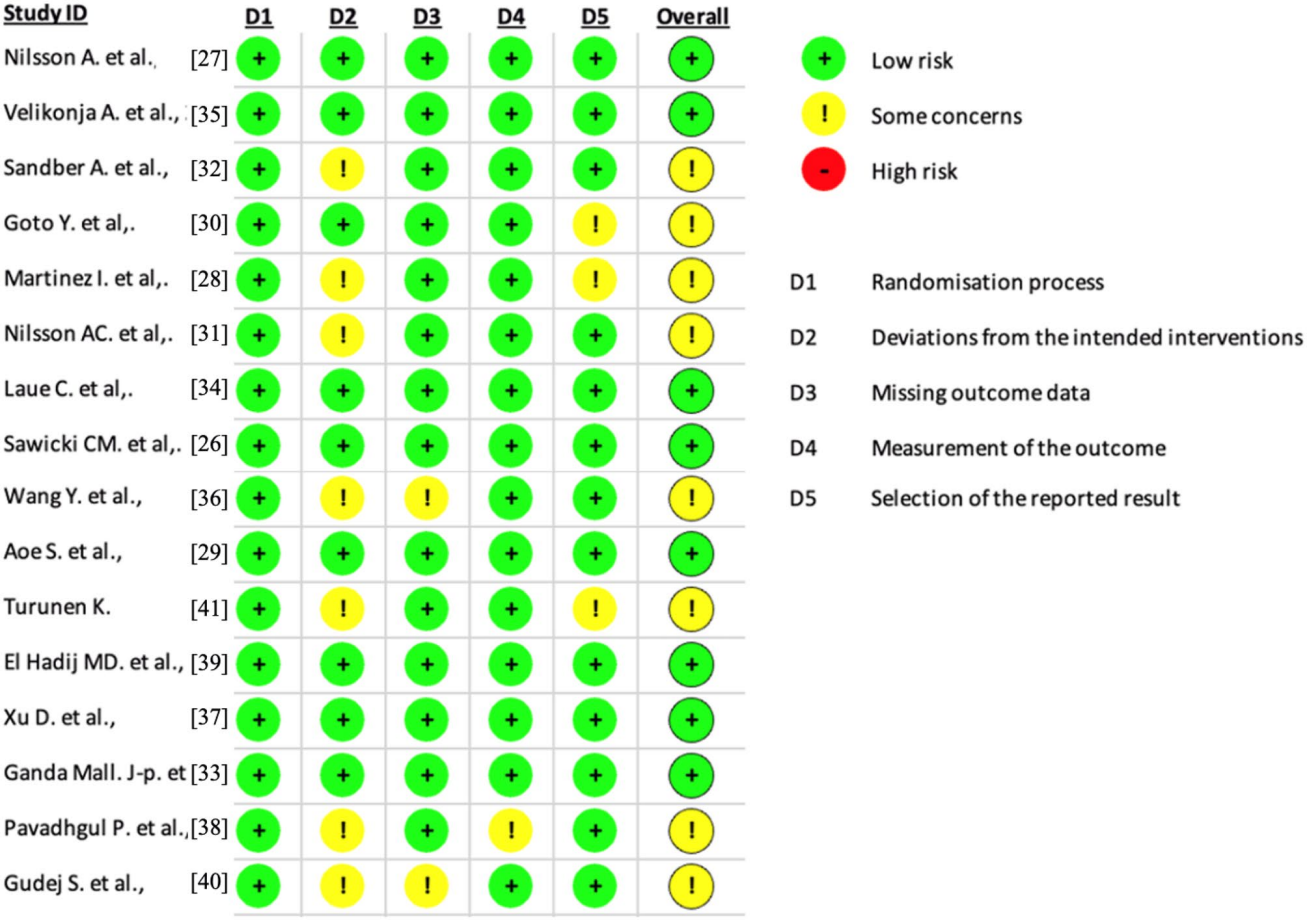
Risk of bias assessment using the revised Cochrane risk-of-bias (RoB 2)

The included studies were assessed against the predetermined criteria of the Cochrane RoB2 tool for randomized control and crossover trials. Within Domain 1: Randomization Process, there were 0 studies with some or high concerns of bias. In Domain 2: Deviations from intended intervention, there were seven with some concern [28, 31, 32, 36, 38, 40, 41] regarding potential bias due to unclear blinding procedures. Specifically, it was uncertain whether sample collectors were aware of the products consumed by participants. This lack of blinding could introduce bias into the study results, as it may influence data collection and interpretation. Two studies had some risk of bias for Domain 3: Missing outcome data, underscoring the importance of evaluating the significance of losses between baseline and final results to avoid underestimating true effects. For Domain 4: Measurement of the outcome, one study [38], demonstrated some risk of bias, highlighting the necessity for consistent measurement methodologies across all participants and interventions to mitigate potential biases. Lastly, in Domain 5: Selection of the reported result, some concerns regarding risk of bias were identified in 3 studies, [28, 30, 41]. It is crucial to ensure consistency between the protocol and reported results, although the absence of a protocol should be acknowledged. Additionally, variability in the presentation of results within tables may introduce bias, emphasizing the importance of adopting standardized methods. All these findings, summarized in Fig. 2, underscore the necessity for meticulous attention to methodological considerations to enhance the reliability and validity of study outcomes.

### Effects of Oats and Barley on the Immune and Inflammatory Outcomes

#### Healthy Young Adults

Three studies were performed in healthy adult population (age range 20–50) who had a BMI < 25 and with no pre-existing conditions [27–29]. Among these, 2 studies measured the effect of whole grain barley consumption on inflammatory biomarkers after consumption of barley derived foods [27, 28]. CRP and IL-6 levels were measured in both studies, and only one measured LBP [28] and the other IL-18 [27]. None of these inflammatory biomarkers showed any level of statistical significance between baseline and endpoint values after consumption of barley products or between the control and intervention groups. It is also important to note, that Nilsson et al. [27] was an acute short-term intervention that mainly evaluated the effects of barley kernel-based products on glucose regulation 14 h after intake. Details of the inflammatory and immune biomarkers from RCTs performed in healthy adults are shown in Table 2.

**Table 2:** RCTs Included in the review performed in healthy and normoweight adults population evaluating inflammatory and immune biomarkers

**Study**	**Intervention diet**	**N**	**IL-6 baseline**	**IL-6 endpoint**	***p* value**
Nilsson et al. (2016) [27]	Barley	C = 21	0.67 ± 0.09	0.95 ± 0.11	NS
		BB = 21	0.90 ± 0.23	1.03 ± 0.19	NS
Martínez et al. (2013) [28]	Barley	BR = 15	1.35 ± 1.36	0.81 ± 0.32	NS
		BR+WGB = 15		0.83 ± 0.38	NS
		WGB = 15		0.86 ± 0.32	NS

**Study**	**Intervention diet**	**N**	**CRP baseline**	**CRP endpoint**	***p* value**
Nilsson et al. (2016) [27]	Barley	C = 21	0.55 ± 0.17	0.55 ± 0.17	NS
		BB = 21	0.42 ± 0.10	0.43 ± 0.10	NS
Martínez et al. (2013) [28]	Barley	BR = 15	1.04 ± 1.93	0.66 ± 0.99	NS
		BR+WGB = 15		0.59 ± 0.83	NS
		WGB = 15		0.94 ± 1.86	NS

**Study**	**Intervention diet**	**N**	**LBP baseline**	**LBP endpoint**	***p* value**
Martínez et al. (2013) [28]	Barley	BR = 15	6.36 ± 6.67	4.83 ± 5.58	NS
		BR+WGB = 15		5.4 ± 5.63	NS
		WGB = 15		5.21 ± 6.83	NS

**Study**	**Intervention diet**	**N**	**Il-18 baseline**	**Il-18 endpoint**	***p* value**
Nilsson et al. (2016) [27]	Barley	C = 21	172 ± 17	162 ± 15	NS
		BB = 21	168 ± 14	173 ± 13	NS

*p* value NS means “No significance”, baseline and endpoint data presented as mean ± S.D (standard deviation)

*N* Number of participants, *CRP *C-Reactive Protein, *IL-6 *Interleukin-6, *LBP* Lipopolysaccharide binding protein, *IL-18* Interleukin-18, *C *Control group, *BB *Barley Bran group, *BR* Brown rice, *WGB* Whole Grain Barley

#### Older Adults

Another population group present in the selected RCTs is the adult population aged 50–80 years old. The study conducted by Laue et al. [34] investigated the adjuvant effects of oat β-glucan on influenza vaccination in healthy seniors, and significant differences in cytokine IFN-γ levels were observed after the oat β-glucan between baseline and one week after vaccination compared to the control group (p < 0.05). However, such differences were not observed in TNF-α, IL-1β, IL-2, IL-12, and IL-10. Similarly, in the study by Ganda et al. [33], there were no significant differences in inflammatory biomarkers after 6 weeks of oat β-glucan extract consumption compared to baseline. In this study, only CRP levels were within the normal range, and interleukins (IFN-γ, IL-10, IL-1 β, IL-2, IL-6, IL-8, TNF-α) were near or below the limit of detection and, therefore, these results were not included. Something similar occurred following a 3-day intervention with a barley kernel-based bread [28], observing no significant differences in serum IL-6 and IL-18 levels after intervention. In the frame of the same study [32], results were analyzed considering two subgroups regarding the abundance of gut *Prevotella* and *Bacteroides*, and a significant effect was detected on the concentrations of IL-6, revealing lower IL-6 concentrations in the higher *Prevotella/Bacteroides* ratio group.

#### Metabolically at Risk Population

Eight studies were included in the review with population with different metabolic risk factors, such as overweight and obesity (BMI = 25–35) [26, 28], metabolic syndrome [35], hypercholesterolemia [36–38] or elevated LDL cholesterol [39]. One of these studies [28] included both normal-weight and overweight participants, but it was also considered here since data were analyzed separately by subgroups. Among these studies, 4 of them measured the effect of barley or oats consumption on inflammatory biomarkers [26, 28, 38, 39]. In the study conducted by Sawicki et al. [26], an acute intake of whole oats or barley compared to refined wheat was performed in overweight and obese populations, and whole grains did not attenuate the post-prandial response of inflammation markers (data not shown in the article).

In the study conducted by Pavadhdul et al. [38] the sustained consumption for 4 weeks of instant whole oat flakes significantly reduced several inflammatory biomarkers such as hs-CRP, IL-6, IL-8, and TNF-α compared to the control product (instant rice flakes) and compared to baseline. Similarly, Martinez et al. [28] evaluated the sustained consumption (4 weeks) of whole grain barley flakes compared to whole grain brown rice or the combination of both, and among the inflammatory markers analyzed (hs-CRP, IL-6 and LBP), only IL-6 was significantly attenuated after consumption of barley products. Despite not achieving statistical significance due to high inter-individual variation, hs-CRP plasma levels were reduced after barley intervention compared with baseline values.

Results of the inflammatory and immune biomarkers from RCTs performed in metabolically at risk population are summarized in Table 3.

**Table 3:** RCTs with population with different metabolic risk factors evaluating inflammatory and immune biomarkers

**Study**	**Intervention diet**	**Risk factor**	**N**	**IL6 baseline**	**IL-6 endpoint**	***p* value**
Martínez et al. (2013) [28]	Barley	Overweight	BR = 13	2.03 ± 1.32	1.64 ± 1.27	NS
			BR+WGB = 13		0.97 ± 0.52	0.0438
			WGB = 13		1.40 ± 0.77	NS
Pavadhgul et al. (2018) [38]	Oat	Hypercholesterolemic	Oat = 24	150 ± 57.9	123 ± 44.5	< 0.01
			Rice = 24		145 ± 54	

**Study**	**Intervention diet**	**Risk factor**	**N**	**CRP baseline**	**CRP endpoint**	***p* value**
Martínez et al. (2013) [28]	Barley	Obese	BR = 13	2.26 ± 2.47	2.12 ± 1.96	NS
			BR+WGB = 13		1.37 ± 1.52	NS
			WGB = 13		1.86 ± 1.87	NS
Pavadhgul et al. (2018) [38]	Oat	Obese	Oat = 24	2.7 ± 2.1	2.2 ± 1.7	< 0.05
			Rice = 24		2.9 ± 2.9	

**Study**	**Intervention diet**	**Risk factor**	**N**	**LBP B baseline**	**LBP endpoint**	***p* value**
Martínez et al. (2013) [28]	Barley	Obese	BR = 13	22.45 ± 24.9	23.56 ± 26.42	NS
			BR+WGB = 13		21.63 ± 23.9	NS
			WGB = 13		22.16 ± 22.66	NS

**Study**	**Intervention diet**	**Risk factor**	**N**	**IL-8 baseline**	**IL-8 endpoint**	***p* value**
Pavadhgul et al. (2018) [38]	Oat	Obese	Oat = 24	286 ± 78.7	229 ± 64.9	< 0.01
			Rice = 24		279 ± 76.7	

**Study**	**Intervention diet**	**Risk factor**	**N**	**TNF-α baseline**	**TNF-α endpoint**	***p* value**
Pavadhgul et al. (2018) [38]	Oat	Hypercholesterolemic	Oat = 24	49.5 ± 26.4	39.83 ± 15.9	< 0.01
			Rice = 24		47.4 ± 24.1	

*p* value NS means “No significance”, baseline and endpoint data presented as mean ± S.D (standard deviation)

*N *Number of participants, *CRP* C-Reactive Protein, *IL-6 *Interleukin-6, *LBP *Lipopolysaccharide Binding Protein, *IL-8* Interleukin-8, *IL-18* Interleukin-18, *TNF-α* Tumor necrosis factor, *B* Barley group, *BR *Brown rice, *WGB * Whole Grain Barley

Another RCT [40] involved individuals diagnosed with chronic gastritis, aged between 23–74, who consumed 100 mL of 3% 1–3,1–4-β-D-glucan from oats. The study analyzed CRP and TNF-α, revealing only a significant decrease in CRP concentration in peripheral blood serum after a 30-day of nutritional intervention, with any significant effect on TNF-α values.

### Analysis of Gut Microbiota Composition and Fecal Metabolites

In this review, 12 articles delve into the exploration of gut microbiota outcomes after barley or oats consumption [27–37, 41]. These studies collectively suggest that barley and oats derived products can influence the composition of gut microbiota, which have been associated in some cases with metabolic changes and improvements, indicating a potential prebiotic effect. The specific effects vary based on dose, type of grain or product (bran, whole grain, β-glucans), intervention duration, and the population studied.

Regarding the studies performed with barley products, in the study conducted by Wang et al. [36] in hypercholesterolemic patients, authors suggest that barley β-glucan induce shifts in gut microbiota in a molecular weight-dependent manner, observing that supplementation of 3 g/day of high-molecular weight β-glucan increased Bacteroidetes and decreased Firmicutes abundances and increased *Bacteroides* at the genus level, whereas diets containing low-molecular weight β-glucan failed to alter the gut microbiota composition.

In another study assessing dietary intervention with barley β-glucans [35], authors observed that after consuming 6 g/day of barley β-glucans during 4 weeks in patients with metabolic syndrome, no significant changes were seen compared to control group at the phylum level for the whole test group, although the production of short chain fatty acids (SCFA) was significantly increased. Interestingly, when participants were grouped according to their response to β-glucans at the cholesterol level, the pre-intervention gut microbiota composition showed higher abundance of health associated *Bifidobacterium* spp. and *Akkermansia municiphila* within cholesterol-responsive group, concluding that barley β-glucans metabolic response is possibly dependent on individual gut microbiota composition. Similarly, Martinez et al. [28] observed that compositional differences at baseline were detected in the gut microbiome of subjects that differed in the magnitude of their anti-inflammatory response to whole grain barley intake. Specifically, subjects with the greatest reduction in plasma IL-6 concentration had significantly higher proportions of *Dialister* and a lower abundance of Coriobacteriaceae*,* suggesting that these taxa may condition the capability of an individual to be immunologically responsive to whole-grain barley intake. Likewise, Sandberg et al. [32] observed that subjects responding to acute barley intake with an improved glucose tolerance tended to have an elevated abundance of *Prevotella* also at baseline prior to intervention.

Aoe et al. [29] tested the effect of the barley bran compared to wheat bran or the interaction of both, and only wheat bran was associated with elevated fecal concentrations of SCFA owing to an increase in the abundance of butyrate-producing bacteria. Additionally, the combination of wheat and barley bran was associated with an increase in the abundance of genus *Bacteroides*, concluding that both wheat alone and wheat combined with barley bran favorably influenced the fecal variables in healthy subjects.

Regarding whole-grain barley interventions, Martinez et al. [28] conducted a 4-week trial with a daily dose of 60 g of whole-grain barley and reported an increase in the microbial diversity, the Firmicutes/Bacteroidetes ratio, and the abundance of the genus *Blautia* in fecal samples. The inclusion of whole-grain barley also enriched the genera *Roseburia*, *Bifidobacterium* and *Dialister*, and the species *Eubacterium rectale*, *Roseburia faecis* and *Roseburia intestinalis.* Similarly*,* Goto et al. [30] assessed the effect of pearled barley intake (which lacks the bran and germ) in healthy subjects during 4 weeks, observing a significant increase in microbial genera that belong to the family Lachnospiraceae, such as *Blautia, Agathobacter*, and *Fusicatenibacter* compared to baseline. Nilsson et al. [27] and Nilsson et al. [31] performed both a short-term intake with whole-grain barley (3-day intervention) in young and middle-aged population, respectively, and analyzed hydrogen levels in expired breath as an indicator of colonic fermentation. In both studies, a significant increase of hydrogen levels after barley interventions was reported, and in the case of Nilsson et al. [31], also an increase of serum SCFA was reported, hypothesizing that an acute intake of barley could rapidly promote a shift in the microbiota composition related to gut fermentation of the dietary fiber fraction in barley grain.

With regard to studies focusing on oats or their β-glucan extracts, Xu et al. [37] demonstrated that consuming 80 g of whole-grain oats, containing 3 g of β-glucans in mildly hypercholesterolemic subjects, significantly increased the abundance of *Akkermansia muciniphila* and *Roseburia*, and the relative abundance of *Dialister*, *Butyrivibrio*, and *Paraprevotella*, bacteria previously shown to protect against metabolic disease. Moreover, *Akkermansia muciniphila*, *Roseburia, Bifidobacterium*, and *Faecalibacterium prausnitzii*, and plasma SCFA correlated with oat-induced changes in plasma lipids, suggesting that the prebiotic activity of oats could contribute towards its cholesterol-lowering effect. When oat β-glucan was administered as an extract with a daily supplementation of 12 g/day in elderly population, no significant effects in the gut microbiota composition were observed after six weeks of intervention [33]. Similarly, Laue et al. [34] assessed the consumption of different non-digestible polysaccharides including oat β-glucans in senior individuals for 5 weeks, and results showed that at the phylum level, the Bacteroidetes/Firmicutes ratio was not significantly different compared to control group. However, significant differences in the abundance of some bacterial genera were noted, such an increase of *Parasutterella* and a non-significant trend towards a lower *Clostridium* relative abundance.

Gudej et al. [40], determined the effect of oat β-glucan supplementation (3 g/day) with low or high molar masses in patients with diagnosed chronic gastritis, and observed that after a 30-day intervention there was a non-significant increase in lactic acid bacteria, whereas, patients consuming both fractions of oat β-glucan experienced a significant increase in SCFA.

## Discussion

The objective of this systematic review was to summarize findings from RCTs assessing the impact of whole-grain barley and oat grains, as well as their isolated fractions, on immune response, inflammatory biomarkers, and modulation of gut microbiota in adult population.

Barley and oat grains exhibit superior functional value, possess the lowest glycemic index, the highest content of β-glucans, resistant starch, and antioxidant properties among all cereal crops. These characteristics suggest that they might be more effective in preventing human diseases and promoting overall health compared to other grains [42, 43]. The anti-inflammatory benefits of whole grains have been shown in various human intervention studies and highlighted in a recent comprehensive systematic review [44••]. Yet, there is currently no comprehensive overview of the literature specifically examining the impact of consuming barley and oats on inflammatory markers from human clinical trials in adults. These grains are particularly intriguing due to their distinct nutritional compositions, and understanding their influence on inflammatory and immune markers, as well as their contribution to the modulation of the gut microbiome, remains an unexplored area.

The present review of 16 RCTs found that the consumption of barley and oats or their individuals grain fractions (bran or endosperm) may have a subtle impact, mildly reducing certain inflammatory markers, including IL-6, IL-8, IL-18, CRP, and TNF-α. Indeed, only 5 out of the 16 reviewed articles noted significant differences, particularly after consumption of whole-grain barley products (bread and flakes) [32, 34] instead of their isolated fractions or extracts. This reduction in inflammatory markers was notably observed in populations metabolically at risk characterized as obese/overweight or with hypercholesterolemia, compared to studies involving healthy subjects, where a trend was observed but without significant differences in outcomes. Importantly, the majority of intervention studies comparing whole grains other than barley or oats with refined grains on pro-inflammatory markers have reported no statistically significant benefits in healthy population [45–47]. Consistent with the findings herein, two previous systematic reviews also concluded that only a significant effect of whole-grain consumption on inflammatory biomarkers was observed in unhealthy individuals with more favorable results within studies with population with metabolic risk factors [44••, 48]. The more favorable results within studies with populations with metabolic pre-existing conditions, are likely due to higher baseline levels of inflammatory markers compared to healthy populations [49–51]. This observation is particularly important because interventions that reduce inflammation are associated with a reduced risk of chronic diseases [52]. It should be noted that in several studies, many of the interleukins measured were near or below the limits of detection. This could indicate that, in the case of healthy individuals, it is more challenging to observe changes in their immune system compared to those with disease, in whom it is easier to detect any complications or inflammation.

Among the 16 studies analyzed in this systematic review, 8 reported the continuation of participants' regular diets without any meal restrictions [27, 29, 33, 35, 37, 39, 41]. This approach allowed participants to maintain their usual dietary habits, potentially exposing them to a variety of dietary factors such as (poly)phenols, fiber, and pro- or prebiotics, which have been shown to exert influences on inflammatory responses and microbiota composition. Furthermore, a subset of studies (4 out of 16) explicitly cautioned against the consumption of prebiotics, probiotics or antibiotics as these directly alter the composition of the intestinal microbiota [31, 32, 34, 41]. This highlights the importance of considering not only the presence but also the potential interactions of these dietary components with barley or oats supplementation. Only a subset of studies (5 out of 16) explicitly noted the importance of avoiding specific dietary components, such as oats, barley, (poly)phenols, and fiber, to prevent potential interference with outcomes related to microbiota composition and inflammatory markers [26, 31, 38–40]. Notably, in the study by Laue et al. [34], participants were instructed to follow a low-fiber diet, underscoring the significance of dietary control in studies investigating the effects of barley or oats supplementation.

Investigating the isolated effects of barley or oats supplementation is crucial for understanding their specific contributions to health outcomes amidst complex dietary patterns. By isolating these effects, researchers clarify the unique impact of barley or oats consumption on physiological parameters, independent of other dietary factors. This approach enhances our understanding of the health benefits of barley or oats supplementation, enabling clearer interpretation of study findings and facilitating targeted dietary recommendations. Isolating the effects also improves research outcomes' reliability and validity, advancing our understanding of their potential role in promoting health and preventing disease.

The present findings underscore the necessity for researchers to carefully consider and control for dietary factors when designing studies examining the effects of barley or oats supplementation. Failure to account for these factors could obscure the true effects of barley or oats, as other dietary components rich in β-glucans or polyphenols may inadvertently influence study outcomes. Therefore, the variation in dietary instructions across studies emphasizes the importance of implementing standardized protocols to minimize variability and enhance the validity of findings concerning the effects of barley and oats on inflammatory responses and microbiota composition in future research.

Furthermore, it is recognized that inflammation is age-dependent, implying that it tends to escalate with advancing age [53, 54]. So, in this review we looked for differences in inflammatory markers at different age groups in order to identify the age ranges in which it is more effective to apply dietary treatment to improve inflammation parameters. Results have shown that in the case of young healthy people, none of the biomarkers of inflammation showed significant differences between baseline and the end of treatment or even between barley or oats consumption and control products. However, in the case of older people, significant differences were observed in some studies. For instance, blood levels of IFN- γ were significantly increased between oat β-glucan (5 week consumption) and control groups in older adults under influenza vaccination (68 years old mean age) [34]. These findings may indicate the potential anti-inflammatory properties of IFN- γ, as previously described [55]. In the case of Sandberg et al. [32], who performed a short-term intervention (3-day) with barley kernel bread in a population between 50 and 70 years old, lower IL-6 concentrations were detected after barley intervention only in the high *Prevotella*/*Bacteroides* ratio group, revealing a main effect of baseline gut microbiota composition.

Previous research has shown the importance of accurately recording the whole grain content of foods in participants diets or, as in our case, the proportion of β-glucan given to participants, rather than by the weight of the food itself, allowing us a more accurate assessment of the dose. Knowing also that the recommended daily dose in adult individuals is between 3 and 4 g of β-glucans daily [56, 57], after analyzing the dose of β-glucan administered to the participants, it was found to be within the recommended dose range in all studies; therefore, it would be good to see if higher doses could lead to significant results.

The role of microbiota in maintaining good health is now clearly established, and the present review suggests that the consumption of oats and barley can support the growth and maintenance of gut microorganisms. Indeed, both the bran and endosperm fractions of oats and barley are rich in dietary fibers, the major source of energy for gut microbiota, and phytochemicals, which can themselves modulate the microbiota. Although oats or barley intake modulated the microbiome in most studies, taxonomic changes indicated high heterogeneity. SCFA production, microbiome diversity, and metabolic-related outcomes varied and did not always occur in parallel with microbiome. Several studies concluded that barley or oats metabolic responses are possibly dependent on individual gut microbiota composition [23, 24, 28, 32]. The fact that pre-intervention microbiota composition is strongly individualized and is an important factor in responses to dietary interventions, has been observed and described in several studies [58, 59].

The increased proportion of Bacteroidetes/Firmicutes genera is an important marker of intestinal dysbiosis and is directly related to recovery from microbiota disorder [35]. Normally, Firmicutes is five times more abundant than Bacteroidetes, but this proportion can be disrupted at some diseases like hypercholesterolemia or obese people [29, 36, 37]. Some studies supported the beneficial role of oats and barley due to an increase in Bacteroidetes over Firmicutes spp. [28, 29, 36], which may be correlated to a reduced risk of coronary heart disease. This microbiota shift was enhanced as the dose of barley increased or as the molecular weights increased [36]. All these could indicate that certain bacteria, such as members of Bacteroidetes, had taken advantage of β-glucan and perhaps other fermentable substrates from oats and barley. The particular role of β-glucans in the growth of Bacteroidetes spp. has been investigated in a study by Tamura et al. [60], showing that a vast majority of humans possess Bacteroidetes spp. capable of utilizing β-glucans, therefore, confirming the prebiotic character of β-glucans.

Some studies also propose that dietary polyphenols can have a prebiotic-like effect, especially those fiber-bound (poly)phenolic compounds, promoting the growth of beneficial bacteria, including Bacteroidetes, and potentially influencing the Bacteroidetes/Firmicutes ratio [24]. However, the effects can vary depending on the type of polyphenol, its concentration, the individual's overall diet, and other factors. It is essential to note that the microbiota is highly individualized, and responses to polyphenols can differ among people [61].

In terms of specific bacterial genus, some studies reported that oats and barley consumption favorably increased the growth of beneficial bacteria involved in saccharolytic and butyrate production which act as potent anti-inflammatory, including *Akkermansia*, *Roseburia*, and *Bifidobacterium* [28, 29, 37, 40]. The fact that *Roseburia* spp. are involved in carbohydrate fermentation is associated with increased acidification of the environment, which is reflected in inhibiting the growth of pathogenic microorganisms, as well as regulating glucose levels to prevent the onset of pre-diabetes and diabetes [62, 63]. *Akkermansia muciniphila*, is linked to a healthy gut, potentially mitigating metabolic syndrome, and its abundance inversely correlates with various pathological states, as it produces SCFA, enhance mucosal thickness and improve the intestinal barrier's function, and therefore, is conceived as a biomarker for healthy gut microbiota [64]. *Akkermansia* was found to be increased after the consumption of barley flakes for 17 weeks in a healthy population [28] and increased in mildly hypercholesterolemic individuals after 5 weeks of barley β-glucan intake [36]. In another study [35] only the participants who had higher abundance of *Akkermansia* *muciniphila* already within the pre-intervention microbiota responded to diet intervention (bread containing 6 g of barley β-glucans) by a reduction in total cholesterol.

SCFA were analyzed in 5 papers [29, 31, 34, 40, 41]. In the case of Nilsson et al. [31], fecal SCFA were increased after 3 days consuming barley kernel bread compared to the control group, indicating a possible improvement at gut microbiota composition. Something similar happened with Gudej et al. [40], who observed an increased on the concentrations of acetic acid, propionic acid and hydroxybutyric acid in patients that consumed oat β-glucan fraction, which was reflected in an increase of fiber-metabolizing bacteria in the gut, such as *Bifidobacterium* and Clostridiaceae. Collectively, this review suggests that including oats or barley in the diet, even for a short period of time, can result in marked changes in the gut microbiota and promote the synthesis of SCFA, which agrees with previous observations performed on animal models [65].

One of the strengths of this review is the organization of the studies. For example, the careful division of barley or oat β-glucan, which although both contain a β-glucan with the same type of linkage, could have some type of different outcomes. In addition, only blood measurements of cytokines and microbiota from fecal samples were included. It should also be noted that an important aspect of this review was not to include articles with multifunctional diets or other types of grains included in the same food that could affect our results due to their composition. Likewise, the collection of data grouped in healthy or diseased individuals allowed the comparison between the different types of populations, highlighting possible differences between them, something to be studied more intensively in future reviews.

Finally, some limitations such as different methods of whole-grain prescription, calorie restriction in several studies, different methods used for measuring inflammatory biomarkers or gut microbiota, lack of control for baseline measures in some studies, and different study designs should be considered.

## Conclusions

In conclusion, the reviewed literature provides a substantial foundation for understanding the multifaceted effects of *Avena sativa* and *Hordeum vulgare* L. on immune and inflammatory function, as well as their influence on gut microbiota. Whole grain oats and barley rich in nutrients, dietary fiber, phytochemical compounds, and a range of (poly)phenols, may have the potential to act in an anti-inflammatory manner specially in metabolically at-risk population, which could help impact chronic disease risk. Additionally, these effects seem to be intricately linked to alterations in gut microbiota composition. The reviewed studies collectively suggest that barley and oats derived products can influence the composition of gut microbiota and promote the synthesis of SCFAs, which have been associated in some cases with metabolic improvements, indicating a potential prebiotic effect. Moreover, microbiota compositional differences at baseline may condition the capability of an individual to be immunologically responsive to barley or oat intake, highlighting that that pre-intervention microbiota composition is strongly individualized and is an important factor in responses to dietary interventions.

It is essential to consider the variations in study designs, populations, and interventions when interpreting the findings. Given the small number of trials included and the disparity in the intervention dose products and health states, the conclusions of this study should be interpreted with caution, and more high-quality, long-term studies are warranted. This study further contributes to increasing current knowledge, pointing to future research considerations, particularly the need to conduct research that discerns potential differences and accurately accounts for the dose of whole grain. Additionally, future research should focus on elucidating the underlying mechanisms behind the observed effects, allowing for a more targeted and personalized approach to dietary interventions with barley and oats or their derived products.

## Data Availability

No datasets were generated or analysed during the current study.
